# The Role of Adipokines in Inflammatory Mechanisms of Obesity

**DOI:** 10.3390/ijms232314982

**Published:** 2022-11-29

**Authors:** Tatiana V. Kirichenko, Yuliya V. Markina, Anastasia I. Bogatyreva, Taisiya V. Tolstik, Yurgita R. Varaeva, Antonina V. Starodubova

**Affiliations:** 1 Petrovsky National Research Center of Surgery, 119991 Moscow, Russia; 2 Chazov National Medical Research Center of Cardiology, 121552 Moscow, Russia; 3 Federal Research Centre for Nutrition, Biotechnology and Food Safety, 109240 Moscow, Russia; 4 Medical Faculty, Pirogov Russian National Research Medical University, 117997 Moscow, Russia

**Keywords:** adipokines, inflammation, cytokines, obesity, macrophages

## Abstract

Adipokines are currently widely studied cellular signaling proteins produced by adipose tissue and involved in various processes, including inflammation; energy and appetite modulation; lipid and glucose metabolism; insulin sensitivity; endothelial cell functioning; angiogenesis; the regulation of blood pressure; and hemostasis. The current review attempted to highlight the key functions of adipokines in the inflammatory mechanisms of obesity, its complications, and its associated diseases. An extensive search for materials on the role of adipokines in the pathogenesis of obesity was conducted online using the PubMed and Scopus databases until October 2022.

## 1. Introduction

Obesity is a chronic disease that affects most systems of an organism, leading to the development of non-infectious diseases such as type 2 diabetes, cardiovascular disease (CVD), non-alcoholic fatty liver disease (NAFLD), hypertension, stroke, various forms of cancer, and mental health problems. In addition, obese people were found to be three times more likely to be hospitalized with COVID-19. According to The World Obesity Federation, it is estimated that one billion people globally will be living with obesity by 2030 [1]. Modern studies aiming to study obesity demonstrate the multifactorial nature of its pathogenesis, including hyperinsulinemia, insulin resistance, hormonal regulation, and chronic systemic inflammation as the most important pathogenetic factors in the development of obesity, its complications, and its comorbidities [2]. 

Adipose tissue is an endocrine organ that not only stores lipids, but also secretes various biologically active substances, such as cytokines, adipokines, chemokines, and hormonal factors that regulate metabolic processes in the organism and affect inflammation and endocrine functions [3,4]. Adipokines produced by adipose tissue are biologically active substances that act like classical hormones. They are cellular signaling proteins that regulate or modulate various biological processes in target organs, including the brain, liver, muscles, heart, blood vessels, pancreas, and immune system [5]. Adipokines are involved in various functions and may influence many different processes, including energy and appetite modulation, lipid and glucose metabolism, insulin sensitivity, endothelial cell function, inflammation, angiogenesis, blood pressure, hemostasis, atherosclerosis development, and metabolic syndrome [6,7]. An increase in white adipose tissue (WAT) in obesity, namely its visceral depot (vWAT), affects the secretion of adipokines and is associated with metabolic (obesity, insulin resistance, type 2 diabetes) and cardiovascular diseases due to the properties of vWAT adipocytes, which include reduced sensitivity to insulin, low angiogenic potential, and increased lipolytic activity [8,9,10]. In addition, the negative effect of visceral adipose tissue is associated with the fact that the mobilization of visceral fat increases the transport of free fatty acids (FFA) to the liver through the portal vein and inhibits insulin signaling [11]. It is assumed that vWAT plays a key role in the pathogenesis of obesity and its complications despite of the fact that the conception of the dominant role of visceral WAT in the development of obesity-related complications is not univocal. In turn, subcutaneous WAT (sWAT) is associated with improved metabolism and insulin sensitivity, since it consists of inducible brown adipocytes, containing a high number of mitochondria, which perform thermogenic functions and burn fat [12].

Adipose tissue consists mainly of adipocytes, preadipocytes, and cells of the innate immune system (monocytes, macrophages, and natural killer cells) and the adaptive immune system (different types of lymphocytes), fibroblasts, and vascular cells. In obesity, the cellular composition of adipose tissue changes in terms of the number, phenotype, and localization of immune, vascular, and structural cells [13]. Adipocyte hypertrophy, hypoxia, and increased cell death occur as a result of an increased accumulation of lipids—in particular, triglycerides—which contributes to increased secretion of pro-inflammatory molecules, including tumor necrosis factor α (TNF-α), interleukins (IL)–IL-6 and IL-8, and MCP-1 by adipocytes and immune cells. As a result, there is a significant increase in the number of macrophages in adipose tissue; this is due to the infiltration of circulating monocytes and immune cells into adipose tissue, as well as to the self-renewal of tissue-resident adipose tissue macrophages (ATM) and the retention of macrophages in tissues [14]. At the same time, the content of macrophages is proportional to the degree of obesity and it is greater in vWAT than in sWAT; this is also consistent with the view that visceral adipose tissue plays a large role in the development of insulin resistance and metabolic diseases [13]. Macrophages in adipose tissue cluster around apoptotic adipocytes in the form of aggregates. Adipose tissue macrophages are the primary source of TNF-α, adipokines, and other pro-inflammatory molecules. The content of macrophages in adipose tissue is directly proportional to such indicators of obesity as body mass index (BMI) and adipocyte size [15]. Adipokines released by either adipocytes or macrophages infiltrating adipose tissue, in response to the increase in fat mass, induce low-grade chronic inflammation, insulin resistance and the development of obesity-associated diseases [16,17]. Adipokines have pro- and anti-inflammatory properties and play an important role in the integration of systemic metabolism with immune function [18]. In persons with normal metabolic status, there is a balance of pro- and anti-inflammatory adipokines. This balance shifts in favor of pro-inflammatory mediators as adipose tissue increases in obesity [19]. Thus, anti-inflammatory adipokines stimulate the expression of anti-inflammatory M2 macrophages and attenuate the expression of pro-inflammatory M1 macrophages [20]. In obesity, ATMs tend to polarize to M1 macrophages under the influence of cytokines, as well as glucose and lipid levels [19]. As a consequence, the pro-inflammatory status of adipose tissue contributes to chronic low-grade inflammation and the development of metabolic disorders associated with obesity [21]. Obesity changes the content of adipokines produced by adipose tissue, so it can be concluded that the obesity-specific adipokine profile may play an important role in the pathogenesis of metabolic disorders associated with obesity [16].

The current review attempted to highlight the key functions of adipokines in the inflammatory mechanisms of obesity, its complications, and its associated diseases and to determine the adipokines most proven in clinical studies to be diagnostic markers or therapeutic targets. An extensive search for materials on the role of adipokines in the pathogenesis of obesity was conducted online using the PubMed and Scopus databases until October 2022. The following keywords were used in different combinations for the literature search: obesity, adipokines, pathogenesis, leptin, resistin, visfatin, chemerin, DPP-4, adiponectin, omentin, isthmin, nesfatin, preparations, and clinical study.

## 2. Pro-Inflammatory Adipokines in Obesity and Its Complications

Adipokines are considered to be regulators of homeostasis throughout the body. To date, more than 600 adipokines with different biological activities have been described. Obesity is associated with the activation of pro-inflammatory adipokines and the development of chronic low-grade inflammation. In turn, the secretion of anti-inflammatory adipokines is suppressed in obesity [22,23]. The pathophysiological effects of the most studied adipokines are described below.

### 2.1. Leptin

One of the best-known pro-inflammatory adipokines is leptin. Leptin is a hormone synthesized mainly in adipocytes; it provides central weight control through a cognate receptor in the hypothalamus, and it is able to reduce appetite by stimulating the hypothalamus with anorexigenic peptides [24,25]. In addition to appetite regulation, leptin has multiple pleiotropic effects on various biological processes, including energy balance, inflammation, physiological function, and behavior [26]. Leptin has multiple beneficial effects at physiological concentrations, and one of the major risk factors for obesity is leptin resistance, which occurs as a result of impaired leptin transport, leptin signaling, or the hypothalamic neural circuit that regulates energy homeostasis [27]. Leptin is characterized by a circadian rhythm with a maximum level occurring at night [28]. Leptin has a molecular weight of 16 kDa and is encoded by the ob gene, which consists of a 4500 bp sequence, and is secreted into the bloodstream as an active compound consisting of 167 amino acids. The secretion of leptin also depends on other hormones [29]. The leptin receptor (LepR) belongs to the family of cytokine receptors and exists in six isoforms. These isoforms share a common leptin-binding domain but differ in their intracellular domains. LepRa, b, c, d, and f are transmembrane receptors; LepRe consists only of an extracellular ligand-binding domain and is a soluble isoform of LepR [30]. The soluble form of the leptin receptor (sOB-R) can bind to circulating leptin and modulate plasma levels of leptin [31]. Binding of leptin to the LepRb receptor (one of the receptor isoforms) activates Janus kinase 2 (JAK2), signal transducer and transcriptional activator 3 (STAT3), extracellular signal-regulated kinase (ERK), mitogen-activated protein kinase (MAPK), insulin receptor substrate (IRS), phosphatidylinositol 3-kinase (PI3K), protein kinase B (Akt), 5’ adenosine monophosphate-activated protein kinase (AMPK), and others [32,33]. It has been shown that leptin promotes pronounced activation of the transcription factor nuclear factor κB (NF-κB), which, in turn, stimulates pro-inflammatory genes, including genes for chemokines such as monocyte chemoattractant protein-1 (MCP-1) [34]. Pro-inflammatory cytokines increase leptin synthesis and release, contributing to chronic inflammation in obesity. Obesity-induced hyperleptinemia stimulates the production of pro-inflammatory cytokines such as TNF-α, IL-6, IL-2, IL-1β, and interferon-γ (IFN-γ) by monocytes and T-helper 1, and also inhibits the production of the anti-inflammatory cytokine IL-4 [35,36]. Inflammation disrupts the signaling pathways triggered by leptin and contributes to the development of leptin resistance. Leptin resistance leads to a decreased signal of satiety since leptin released by adipocytes due to food consumption tends to reduce eating behavior. A number of factors may also contribute to leptin resistance, including IL-6 production, an increase in C-reactive protein (CRP), and the expression of cytokine signaling suppressor 3 (SOCS3), which exacerbates inflammation and inhibits the activity of the JAK2/STAT3 signaling pathway [37].

Leptin is considered as a potential marker of obesity and its comorbidities—in particular, type 2 diabetes and CVD—due to its angiogenic and atherogenic effects [38]. Several studies have shown that leptin levels are positively correlated with BMI, waist circumference, hip circumference, and insulin resistance parameters [39,40]. It has been shown that leptin level increases not only with increasing BMI, but also depends on gender; in particular, women had significantly higher levels of leptin than men [41]. Genotyping of leptin and the leptin receptor gene can be used to predict insulin resistance and gestational diabetes during pregnancy [42]. In addition, a negative correlation of leptin with high-density lipoprotein (HDL) cholesterol has been shown in healthy subjects [39]. Elevated leptin correlates with atherosclerosis and is an independent predictor of carotid intima-media thickness (cIMT) progression [12,43]. It is known that monocytes/macrophages play an important role in the development of atherosclerosis [44,45,46]. Leptin has been found to be a powerful chemoattractant of monocytes/macrophages and a promoter of their migration, which plays an important role in the development of obesity and concomitant diseases. Leptin facilitates the differentiation of monocytes into macrophages and accelerates the development of cardiovascular complications such as atherosclerosis [47]. Hyperleptinemia contributes not only to increased secretion of inflammatory cytokines, but also to oxidative stress, by increasing the formation of reactive oxygen species (ROS), which causes endothelial dysfunction and also contributes to the development of atherosclerosis. In addition, leptin can induce impaired lipid metabolism [48]. Vascular dysfunction caused by hyperleptinemia has been associated with leptin-mediated downregulation of peroxisome proliferator-activated receptor gamma (PPARγ), which is a key regulator of macrophage lipid metabolism [49,50]. Leptin can stimulate the proliferation and migration of vascular smooth muscle cells (VSMCs), which play a significant role in the formation and development of vascular lesions [51]. It has also been noted that leptin promotes the calcification of cells derived from vascular tissue, which is also of great interest given the importance of vascular calcification in CVDs, particularly in atherosclerosis [52]. A number of studies describe the prothrombotic activity of leptin, which manifests through the stimulation of platelet aggregation and an inhibitory effect on coagulation and fibrinolysis, which may be associated with the stimulation of protein kinase C, phospholipase Cγ2 and phospholipase A2 [43]. Thus, leptin is one of the most well-known and widely studied pro-inflammatory adipokines involved in the development of obesity and its complications.

### 2.2. Resistin

Resistin is an adipokine playing an important role in inflammation and energy homeostasis, and is involved in the development of obesity, insulin resistance, and comorbidities, particularly atherosclerosis. It is encoded by the RETN gene [53,54,55]. Resistin is a polypeptide with a molecular weight of 12.5 kDa [56]; it consists of 108 amino acids, its secretion is increased with obesity, and it acts directly on adipocytes, inhibiting insulin-induced glucose uptake. It has been shown that resistin is highly expressed in bone marrow and macrophages in humans [57]. It is produced mainly by monocytes/macrophages and, in small amounts, directly by adipocytes, thereby modulating the molecular pathways involved in inflammatory, metabolic, autoimmune, and cardiovascular diseases. Resistin is involved in angiogenesis, thrombosis, migration, and the proliferation of VSMC; moreover, it affects endothelial dysfunction, which contributes to the development of atherosclerosis [58]. In macrophages, resistin has been shown to modulate the PPARγ-dependent PI3K/Akt signaling pathway in the activation of sterol-regulatory element-binding proteins (SREBPs) involved in cholesterol metabolism [59]. The action of resistin is attenuated by PPARγ agonists [60]. The pro-inflammatory effect of resistin may be mediated by binding to Toll-like receptor 4 (TLR4) and the subsequent activation of NF-κB in macrophages via the c-Jun N-terminal kinase (JNK) and p38 MAPK pathways; these contribute to the development of insulin resistance and inflammation, closely related to obesity and associated metabolic diseases [61,62]. It has been demonstrated that resistin levels are significantly higher in overweight and obese patients, but have not been found to correlate with cIMT [63]. In addition, it has been shown that resistin levels are positively correlated with insulin resistance in people with hyperresistinemia, in contrast to people with normal levels of circulating resistin in obesity and T2DM [64]. Thus, we can conclude that resistin is involved in the development of obesity and its vascular complications, particularly atherosclerosis.

### 2.3. Visfatin

Visfatin is a pro-inflammatory adipokine that is presumably produced and secreted predominantly by vWAT. It has a molecular weight of 52 kDa and consists of 473 amino acids [65]. Visfatin is expressed and released by macrophages of vWAT in response to inflammatory signals [66]. Visfatin (nicotinamide phosphoribosyltransferase (NAMPT)) exists in two forms: intracellular (iNAMPT), which plays an important role in nicotinamide adenine dinucleotide (NAD+) biosynthesis, and extracellular (eNAMPT), associated with many hormone-like signaling pathways and intracellular signaling cascades [67]. The pro-inflammatory effects of visfatin are associated with the activation of the transcription factor NF-κB, activator protein 1 (AP-1), cytokines IL-6, IL-8, and TNF-α, adhesion molecules such as intercellular adhesion molecule-1 (ICAM-1), vascular cell adhesion molecule-1 (VCAM-1), E-selectin, and (MMP)–MMP-1 and MMP-3 [68]. Moreover, the ability of visfatin not only to increase the level of TNF-α, but also to respond to the stimulation of TNF-α with increased expression, which may represent a pathogenic loop, was revealed [69,70]. This is an important mechanism in the development and progression of inflammatory diseases, including both obesity and its vascular complications, particularly, atherosclerosis. It has been demonstrated that visfatin expression is associated with instability of atherosclerotic plaques since it was significantly enhanced in symptomatic carotid atherosclerosis compared with plaques from asymptomatic individuals; additionally, increased visfatin content was demonstrated in ruptured plaques of patients with acute myocardial infarction [69]. In addition, visfatin increases the expression of vascular endothelial growth factor (VEGF) by activating the PI3K/Akt, ERK1/2, p38 MAPK, and JNK1/2 signaling pathways, which induces endothelial angiogenesis [71]. It has been shown that an increase in the level of visfatin is associated with an increase in cIMT, so it can be considered a marker of subclinical atherosclerosis [72].

### 2.4. Chemerin

Chemerin is an adipokine with a molecular weight of 14 kDa, involved in various physiological and pathophysiological processes, the regulation of adipogenesis, insulin sensitivity, and immune response, and is increased in obesity [73]. Chemerin is expressed by several receptors, including two receptors with signaling activity–G-protein-coupled chemokine-like receptor 1 (CMKLR1) and G-protein-coupled receptor 1 (GPR1)—and chemokine-like receptor-like 2 (CCRL2) with atypical signaling activity [74]. Chemerin and CMKLR1 are expressed in adipose tissue, with chemerin predominantly in adipocytes, and CMKLR1 both in adipocytes and in the stromal vascular cells of adipose tissue [75]. Circulating chemerin level is elevated in inflammatory conditions and has been shown to be activated by pro-inflammatory cytokines TNF-α, IL-1β, and IL-6. It has been demonstrated that chemerin activates the pro-inflammatory pathways PI3K/Akt and MAPKs [76]. Chemerin also increases the expression and secretion of endothelial cell adhesion molecules VCAM-1, ICAM-1, and E-selectin, which leads to increased monocyte–endothelial adhesion, a critical step in the development of atherosclerosis [77]. Chemerin was positively correlated with BMI, C-reactive protein, and the homeostasis model assessment of insulin resistance (HOMA-IR) index and negatively correlated with HDL levels [78]. Thus, chemerin is an important marker of inflammatory processes accompanying the development of obesity and its complications.

### 2.5. DPP4

Dipeptidyl peptidase 4 (DPP4) can also be considered an adipokine secreted primarily by visceral adipose tissue, where it exhibits autocrine and paracrine activity. It is a widely expressed serine protease that regulates the biological activity of many peptides through cleavage and inactivation, including incretin hormones, glucagon-like peptide-1 (GLP-1), and glucose-dependent insulinotropic polypeptide (GIP) [79]. DPP4 is a pro-inflammatory adipokine that is elevated in several metabolic diseases and can impair insulin signaling in adipocytes, as well as other target cells and tissues [80]. In obesity, DPP4 serum concentration was associated with increased macrophage infiltration of vWAT, high serum levels of leptin, and reduced adiponectin levels, as well as increased circulating levels of inflammatory cytokines, particularly IL-6, IL-12, and TNF-α [81]. A positive correlation was found between circulating DPP4 and TNF-α and IL-1β levels [82]. It has been demonstrated that the plasma level of DPP4 is increased in obese patients with metabolic syndrome [83]. DPP4 was shown to contribute to the development of insulin sensitivity through a reduction in Akt phosphorylation [84]. DPP-4 is an important marker of obesity and its complications, particularly diabetes mellitus, which is actively used as a target for therapy and the development of drugs that reduce blood glucose levels.

**Figure 1:**
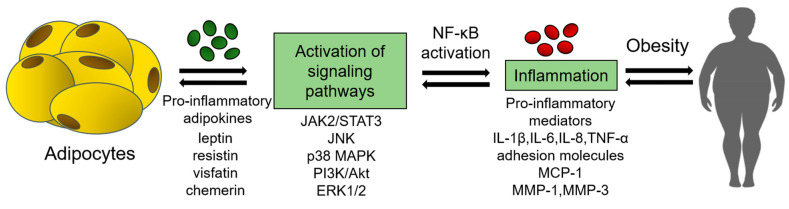
Pro-inflammatory effects of adipokines. ERK1/2—extracellular signal-regulated kinase 1/2; IL—interleukins; JAK2/STAT3—Janus kinase 2/signal transducer and transcriptional activator 3; JNK—c-Jun N-terminal kinase; MCP-1—monocyte chemoattractant protein-1; MMP—matrix metalloproteinases; NF-κB—nuclear factor κB; p38 MAPK—p38 mitogen-activated protein kinase; PI3K/Akt—phosphatidylinositol 3-kinase/protein kinase B; TNF-α—tumor necrosis factor α.

The pro-inflammatory effects of the most studied adipokines are shown in Figure 1.

## 3. Anti-Inflammatory Adipokines in Obesity and Its Complications

### 3.1. Adiponectin

Adiponectin is an adipokine secreted by WAT that exerts pleiotropic effects on numerous physiological processes [85]. Adiponectin is a protein with a molecular weight of about 30 kDa that consists of 244 amino acids [86]. It is known as an anti-inflammatory, anti-diabetic, and anti-atherosclerotic adipokine, acting through the AdipoR1 and AdipoR2 receptors [87]. Adiponectin increases insulin sensitivity and fatty acid oxidation, and reduces hepatic glucose production [88]. Binding to its receptors, adiponectin initiates a signaling cascade, including the phosphorylation of AMPK and p38 MAPK, and also increases peroxisome proliferator-activated receptor α (PPARα) ligand activity [89]. The adiponectin-mediated activation of AMPK, as well as the activation of endothelial nitric oxide synthase (eNOS), stimulates the production of nitric oxide in endothelial cells, which has a positive effect on endothelial function and the maintenance of vascular tone [90]. The pro-inflammatory effect of adiponectin is also manifested in the inhibition of the TNF-α-induced expression of endothelial adhesion molecules, the suppression of inflammatory cytokine production, NF-κB activation, and the proliferation and migration of VSMC [91,92,93]. In addition, adiponectin has been shown to be a macrophage polarization regulator; it stimulates the expression of M2 macrophages, and attenuates the expression M1 macrophages, thereby helping to reduce inflammation [20]. 

Low levels of adiponectin are associated with increased levels of risk factors for cardiovascular disease and markers of oxidative stress. It has been found that increased circulating levels of adiponectin positively correlate with serum HDL cholesterol, the ratio of total cholesterol/LDL cholesterol, and antioxidant capacity, and is inversely associated with the HOMA-IR insulin resistance index and lipid peroxidation in serum [94]. Moreover, an inverse correlation between circulating levels of adiponectin and BMI has been shown [95]. However, some differential associations have been identified between adiponectin levels and fat accumulation in the trunk and lower extremities. Thus, the accumulation of body fat is inversely correlated with the level of adiponectin, while the accumulation of fat in the lower extremities is associated with a higher level of it [96,97]. In addition, adiponectin levels correlated negatively with cIMT [98]. Thus, adiponectin is the most studied anti-inflammatory adipokine, and can be used as a diagnostic marker of obesity, as well as the development of concomitant diseases.

### 3.2. Omentin

Omentin is an adipokine with anti-inflammatory potential that is expressed and secreted by visceral adipose tissue. Omentin is a protein with a molecular weight of 35 kDa consisting of 313 amino acids. Two isoforms of omentin are known, and omentin 1 is the main one circulating in human blood [99,100]. Decreased omentin levels have been observed in obesity, insulin resistance, and type 2 diabetes, as well as in cardiovascular diseases such as atherosclerosis and other inflammatory diseases, particularly inflammatory bowel diseases [101,102,103].

The anti-inflammatory effect of omentin is associated with its inhibitory effect on the expression of components of inflammatory signaling pathways. Thus, it has been shown to inhibit the expression mRNA and TLR4 protein, as well as NF-κB phosphorylation [104]. It also has been found that omentin-1 promotes phosphorylation of the signaling pathway associated with integrin, as well as Akt and AMPK in macrophages, and suppresses the expression of inflammatory cytokines [105]. The concentration of omentin-1 is inversely related to the levels of pro-inflammatory cytokines such as IL-6 and TNF-α [106]. A positive effect of omentin on endothelial function through improvement of the production of nitric oxide [107], which reduces oxidative stress and apoptosis [108], has been demonstrated.

It has been shown in some studies that circulating omentin levels are independently and negatively correlated with cIMT in healthy and diabetic subjects [109,110]. Omentin can be considered a potential biomarker for atherosclerosis and CVD. Thus, it has been demonstrated that decreased omentin levels were observed in patients with atherosclerotic acute cerebral infarction, and a negative relationship has been shown between the unfavorable prognosis of this disease and omentin levels [111].

### 3.3. Isthmin 1

Isthmin 1 (ISM1) has more recently been identified as an adipokine secreted by adipose tissue, and plays a role in increasing glucose uptake by adipose tissue while suppressing hepatic lipid synthesis [112]. It belongs to the isthmin gene family, which includes ISM1 and ISM2. The ISM1 gene is located on chromosome 20 and encodes a ~60 kDa protein containing 499 amino acids [113]. Isthmin 1 signaling is PI3K-dependent and shares phosphorylation targets with insulin signaling; these most likely occur via yet-unidentified receptor tyrosine kinase but not via the insulin receptor or insulin-like growth factor 1 receptor [114]. Studies have shown that ISM1 possesses an anti-inflammatory effect, probably by suppressing NF-κB activation and the production of inflammatory cytokines/chemokines [115]. It has been also found that ISM is an inducer of endothelial cell (EC) apoptosis and an inhibitor of angiogenesis [116].

### 3.4. Nesfatin 1

Despite the fact that nesfatin-1 is a peptide secreted by peripheral tissues in the central and peripheral nervous system, it plays an important role in the system regulation of energy homeostasis [117]. Nesfatin 1, as well as its precursor protein nucleobindin-2 (NUCB2), are potent anorexigenic peptides, which physiologically inhibit food intake and body weight [118]. The involvement of nesfatin in carbohydrate metabolism is confirmed by the fact that it stimulates the expression of pre-proinsulin mRNA, increases glucose-induced insulin release, and also inhibits glucagon secretion [119]. It has been found that nesfatin increases insulin sensitivity and has anti-inflammatory as well as cardioprotective effects. Thus, studies have shown that nesfatin inhibits MAPK signaling pathways, including p38 MAPK/c-Jun and IKK/NF-κB, and also reduces the levels of inflammatory cytokines IL-6, IL-1β, and TNF-α [120,121,122]. In addition, it has been found that nesfatin affects indicators of oxidative stress, namely, it suppresses the increased production of ROS, and also increases the activity of superoxide dismutase [122]. Thus, nesfatin may be a biomarker for metabolic and cardiovascular diseases [123].

## 4. Adipokines in Clinical Trials

### 4.1. Adipokines as Prognostic Markers

The most studied adipokines are successfully included in the design of clinical trials as predictive markers of various obesity-related diseases, as well as endpoints in studies of the efficacy of preparations. Numerous studies demonstrate the association of physical activity, lifestyle correction, and weight loss with different adipokine levels [124,125,126]. However, in prospective population-based study of 6.502 participants free of CVD at baseline, no association of adiponectin and leptin levels with cardiovascular outcomes was revealed after a mean follow-up time of 11.4 years [127]. Table 1 presents the results of clinical trials demonstrating the association of some adipokines with obesity, coronary artery disease, diabetes, NAFLD, and arterial hypertension.

Studies have shown a change in the profile of adipokines in obese individuals with impaired glucose tolerance in response to weight loss as a result of prolonged exercise and diet. So, the level of leptin in the serum significantly decreased, and the concentration of adiponectin in the serum increased [140,141]. The meta-analysis data demonstrate that after bariatric surgery, accompanied by an improvement in fat mass, circulating levels of adipokines changed as follows: adiponectin increased significantly, leptin and chemerin levels decreased, and visfatin and resistin levels did not change [142].

### 4.2. Adipokines as Therapeutic Targets 

Numerous therapeutic agents have been studied as preparations modulating adipokines’ circulating levels in obesity and associated diseases. Table 2 summarizes the effects of the most studied therapeutic agents on adipokine concentrations in different models.

Many studies demonstrate the beneficial effect of anti-diabetic preparations on serum concentrations of adipokines, which correlate with indicators of their anti-inflammatory activity. It has been shown in a systematic review that metformin in patients with metabolic syndrome downregulated leptin, visfatin, and resistin levels while increasing adiponectin concentration [143]. In diabetic patients, the ameliorating effect of metformin on the adiponectin/leptin ratio was concomitant with the reduction in inflammatory cytokines IL-6 and TNF-α [144]. Another widely used group of preparations in the treatment of type 2 diabetes is sodium glucose-like transporter 2 inhibitors (SGLT-2). The meta-analysis dedicated to reviewing the influence of sodium–glucose cotransporter-2 inhibitors on plasma adiponectin levels revealed significantly increased plasma adiponectin in type 2 diabetic patients administered tofogliflozin, luseogliflozin, and ipragliflozin, but not dapagliflozin [145]. In another study, dapagliflozin was shown to downregulate circulating leptin levels in patients with type 2 diabetes and class 3 obesity during a 1-year administration [146]. Vildagliptin, a DDP-4 inhibitor used in therapy for type 2 diabetes, has been shown to increase adiponectin levels, as well as to improve inflammatory biomarkers and LDL oxidation in obese diabetic patients [147]. Preparations of the thiazolidinedione family’s activators of PPARγ improved adiponectin levels while possessing a neutral effect on leptin levels in several studies including participants with NAFLD, nonalcoholic steatohepatitis, and type 2 diabetes; however, their clinical use was limited by long-term side effects such as increased risk of myocardial infarction [148]. Pioglitazone, a drug from the thiazolidinediones family, has been also shown to upregulate circulating omentin-1 levels [149].

A meta-analysis of randomized controlled trials demonstrated that treatment with n-3 polyunsaturated fatty acids (PUFAs) significantly increases circulating adiponectin in patients with type 2 diabetes [150]. A number of studies have also studied the effect of probiotic-based preparations on basic adipokines. For example, preparations based on *Lactobacillus* ameliorated plasma concentrations of leptin in several studies of obese patient [151]. However, a recent systematic review of probiotic intervention in patients with prediabetes and type 2 diabetes mellitus reported no significant alterations in adiponectin and leptin levels [152].

Different groups of preparations used for CVD treatment may influence the serum concentrations of adipokines. Firstly, statins and 3-hydroxy-3-methylglutaryl coenzyme A reductase inhibitors should be described since their pleiotropic anti-inflammatory effects seem to be promising in regard to the improvement of serum adipokines. A systematic review of the effects of atorvastatin, simvastatin, rosuvastatin, pravastatin, and pitavastatin on adiponectin demonstrated a significant increase in plasma adiponectin levels following statin therapy [153]. However, the latest meta-analysis of randomized controlled trials reported that atorvastatin treatment did not significantly affect circulating adiponectin [154]. Moreover, multiple studies dedicated to investigation of the statin-induced amelioration of serum adipokines demonstrated either controversial (adiponectin) or non-significant (leptin and resistin), and rare significant effects (visfatin) [155]. Telmisartan, a preparation from the group of angiotensin II receptor blockers widely used for blood pressure correction, significantly increased adiponectin levels in patients with arterial hypertension combined with obesity [156], while perindopril and angiotensin-converting enzyme inhibitor demonstrated the most prominent action on serum leptin reduction among other anti-hypertensive preparations [157].

**Table 2:** Preparations modulating circulating adipokine levels.

Intervention	Study Design	Effects
Metformin [143,144]	Individuals with metabolic syndrome without restriction in age treated with a dose of 500–3000 mg/day for 3–12 months (several studies included in systematic review)	↑ adiponectin ↓ leptin ↓ resistin ↓ visfatin
Dapagliflozin [146]	Patients with type 2 diabetes and class 3 obesity, 10 mg/day over a 12-month treatment period	↓ leptin n/a adiponectin
Vildagliptin [147]	Women with obesity and type 2 diabetes, 50 mg twice/day over a 30-day treatment period	↓ DPP4 ↑ adiponectin
Pioglitazone [148]	Patients with nonalcoholic steatohepatitis, 45 mg/day with 6 months of administration	↑ adiponectin n/a leptin
PUFAs [150]	Patients with type 2 diabetes, >8 weeks of treatment	↑ adiponectin ↓ leptin
*Lactobacillus* probiotic [151]	Subjects with BMI of 25–30 kg/m^2^ on a diet including 4 × 109 colony-forming units of *L. plantarum* for 12 weeks	↓ leptin
Statins [153]	Studies included in systematic review, statin administration for >2 weeks at the following doses: atorvastatin, 10–80 mg/day; simvastatin, 10–40 mg/day; rosuvastatin, 2.5–10 mg/day; pravastatin, 10–40 mg/day; pitavastatin, 2 mg/day	↑ adiponectin
Telmisartan [156]	Patients with arterial hypertension combined with obesity, 80 mg/day, 12-week treatment period	↑ adiponectin
Perindopril [157]	Overweight or obese patients with hypertension, 10 mg/day, 24-week treatment period	↓ leptin

n/a, not affected; DPP4, dipeptidyl peptidase 4; PUFAs, n-3 polyunsaturated fatty acids.

## 5. Conclusions

Summing up this review, it should be noted that the concept of adipokines implies many different biologically active compounds that make a significant contribution to the key stages of inflammation, metabolic regulation, and the development of obesity and its complications. The term adipokines includes numerous biologically active substances produced by adipose tissue that regulate metabolic processes; therefore, many of them were not described in this review, as they were previously characterized in detail, perticularly inflammatory cytokines and other mediators. Currently, leptin, resistin, and visfatin are the most widely used pro-inflammatory adipokines in experimental studies, as well as in clinical trials of anti-diabetic preparations and other therapeutics targeting obesity-associated diseases. At the same time, adiponectin, considered an anti-inflammatory adipokine, is the most studied among all known adipokines, especially as a marker of the anti-inflammatory efficacy of various preparations, as well as in research aimed at investigating the pathogenesis of obesity and its complications. Thus, adipokines may be included in novel diagnostic strategies as biological markers of various metabolic, inflammatory, and cardiovascular diseases, since numerous studies demonstrate their association with traditional risk factors, clinical characteristics, and the severity of symptoms of obesity and its associated disorders. The results of recent studies allow some adipokines to be considered as promising targets for the treatment of obesity and the prevention of its complications.

## Figures and Tables

**Table 1:** Association of adipokine levels with obesity and associated diseases.

Obesity-Associated Diseases	Study Design	Results
Obesity	Study of the adiponectin/leptin ratio (AdipoQ/Lep) as a biomarker of adipose tissue and metabolic function in obese individuals without diabetes.	Lower AdipoQ/Lep correlated with higher BMI, body fat mass, waist-to-height ratio, and plasma resistin and may, therefore, be an early marker for the development of insulin resistance in obese adults [128].
	Study of resistin levels in obese Nigerian Africans.	The resistin concentration was higher in obese individuals [129].
	Study in control vs. obese women at baseline and after 12 weeks of following the Mediterranean diet.	Serum leptin levels were higher in obese women and weight loss was associated with a decrease in serum leptin [125].
Coronary artery disease	Study of patients with coronary artery disease with isolation of adipocytes from subcutaneous, perivascular, and epicardial adipose tissue.	Decreased expression and secretion of the adiponectin gene in epicardial adipose tissue, and high level of leptin gene expression [130].
	Study of adipokine levels in individuals with coronary artery calcium progression.	Higher leptin levels were associated with coronary artery calcium progression; no association was observed for resistin and adiponectin [131].
	Study of anthropometric and echocardiographic parameters and adipokine levels in Caucasian patients.	Adiponectin and resistin, but not leptin, were associated with echocardiographic parameters of cardiac remodeling and dysfunction [132].
NAFLD	Study of adipokine levels in obese/overweight children and their association with the degree of hepatosteatosis.	Levels of leptin, omentin-1, and adiponectin were higher in the obese group and increased with a higher degree of hepatosteatosis [133].
	Study of adipokines in patients without NAFLD, with steatosis and with NAFLD.	Lower plasma levels of adiponectin were associated with the presence and severity of NAFLD [134].
Diabetes	Study of the factors associated with the onset of type 2 diabetes, including serum adiponectin.	In women, unlike men, a decrease in the level of adiponectin was the only significant risk factor for the development of type 2 diabetes [135].
	Study of the level of leptin as a biomarker for the development of insulin resistance in patients with type 2 diabetes.	Higher serum leptin levels were found in patients with type 2 diabetes and metabolic syndrome [136].
	Study of the relationship of adipokines in patients with type 2 diabetes in Saudi Arabia.	The decrease in adiponectin levels and the increase in leptin, visfatin, and chemerin were more significant as BMI increased in patients with type 2 diabetes; no association of resistin with type 2 diabetes was found [41].
Arterial hypertension	Study of resistin levels in patients with and without arterial hypertension.	The level of resistin did not differ significantly between patients with and without arterial hypertension [137].
	Study of the adiponectin–resistin index as a marker of hypertension in obese patients.	The adiponectin–resistin index was strongly associated with an increased risk of obesity-related hypertension [138].
	Study of the relationship between leptin levels and arterial hypertension as a complication of type 2 diabetes mellitus.	Leptin levels in patients with arterial hypertension and in patients with type 2 diabetes were higher than in the group without hypertension [139].

## Data Availability

Not applicable.
